# Dataset of DDoS attacks on Fibaro home center 3 for smart home security

**DOI:** 10.1016/j.dib.2024.110991

**Published:** 2024-10-03

**Authors:** Ladislav Huraj, Marek Šimon, Jakub Lietava

**Affiliations:** Institute of Computer Technologies and Informatics, University of Ss. Cyril and Methodius in Trnava, 91701 Trnava, Slovak Republic

**Keywords:** Network traffic, SYN flood, ICMP flood, HTTP flood, Cyber security, DDoS

## Abstract

DDoS attacks pose a significant security risk to smart homes and can disrupt the functionality and availability of connected devices in the home. This dataset documents Distributed Denial of Service (DDoS) attacks against the Fibaro Home Center 3 central control unit, which is used to automate smart homes within the Internet of Things. The focus is on three types of DDoS attacks: TCP SYN flood, ICMP flood and HTTP flood. Data collection was performed on the local network, where SYN flood and ICMP flood attacks were performed using the hping3 tool, and HTTP flood attack was performed using the LOIC tool. The data was captured using Wireshark software and is available in PCAP and CSV formats, allowing detailed analysis of the network traffic. The logs include information such as timestamps, source and destination IP addresses, protocols, packet lengths, and port numbers. The dataset includes raw and anonymized data for each type of attack.

The dataset is a resource for researchers focused on cybersecurity and IoT device protection. It allows simulation and analysis of DDoS attacks on a specific IoT device, providing insight into attack patterns and the effectiveness of defenses. The simplicity and specialization of the dataset makes it a practical resource for developing and testing intrusion detection systems and predictive models to mitigate and prevent DDoS attacks. The use of the PCAP format facilitates the import of the data into various research software platforms.

Specifications Table

**Table:** 

Subject	Computer Science: Computer Networks and Communications, Cryptography and Cybersecurity
Specific subject area	DDoS attacks to exhaust the resources of a specific IoT device Fibaro Home Center 3, TCP SYN flood, ICMP flood and UDP flood.
Type of data	Raw, Anonymised, Pcap files
Data collection	The flood packets for the SYN flood attack and for the ICMP flood attack were generated by two computers using the hping3 tool, and the LOIC tool was used for the HTTP flood attack. The attacks were performed on the Fibaro Home Center 3 unit and logged using Wireshark software. During the attack, a mobile phone was connected to the infrastructure to monitor the functionality of the mobile application and the alerts from the sensors of the Fibaro Home Center 3 unit during the execution of the attacks. The resulting dataset includes PCAP and CSV files for each type of attack performed.
Data source location	University of Ss. Cyril and Methodius in Trnava, Trnava, Slovakia
Data accessibility	UCM_FibIoT2024 Repository name: Mendeley Data identification number: 10.17632/p42xjtv8pv.1 Direct URL to data: https://data.mendeley.com/datasets/p42xjtv8pv/1
Related research article	none

## 1 Value of the Data

• The dataset includes three basic flood attacks on the Fibaro Home Center 3 smart home control unit, which enables the connection of smart devices. The dataset also includes control communications with the Fibaro Center to verify IoT functionality during specific types of flood attacks.
• Information from the dataset can be used to develop new or improved defense strategies against DDoS attacks for IoT devices, contributing to the security of the smart home.
• Unlike other available DDoS datasets targeting IoT devices [[1], [2], [3]], where it is difficult to track a specific type of attack on a specific IoT device, this dataset is simple and specialized to only one IoT device. The dataset allows for easier simulation and analysis due to the smaller number of packets compared to other datasets [[1], [2], [3]]. In addition, the sequential order of attacks allows for a more thorough study of attack patterns and responses, as well as attack dynamics.
• Researchers can use the dataset to test and validate new intrusion detection systems or to develop predictive models to predict and prevent attacks. The dataset's pcap format facilitates data import into a variety of research software platforms.

## 2 Background

A smart home is built on the Internet of Things (IoT), where the interconnection of IoT devices, data collection and management in a smart home is typically managed by a central unit. The central unit, which is part of the smart home, is an important element for the efficient functioning of the smart home as it enables the integration and coordination of different devices and services. In addition, many users prefer to access the central unit directly rather than using cloud services [[4], [5], [6]]. The central control unit, like other IoT devices in the smart home, is vulnerable to DDoS attacks [7].

The motivation for compiling this dataset was to provide a dedicated data source that would facilitate the simulation and analysis of DDoS attacks on a specific central control unit. The theoretical and methodological background includes research in the area of cybersecurity of IoT devices [8], with the goal of improving the understanding and identification of DDoS attack characteristics and thus contributing to the development of effective defense mechanisms. The dataset contains data from testing DDoS attacks such as SYN flood, ICMP flood and HTTP flood on the Fibaro Home Center 3 central unit. The advantage of the dataset is its simplicity and specialization, which may appeal to researchers and experts in the field of cybersecurity, enabling them to analyze and develop defense mechanisms more efficiently.

## 3 Data Description

The UCM_FibIoT2024 dataset consists of network traffic samples recorded during a DDoS attack on a central control unit in a smart home. The dataset includes three basic types of DDoS attacks: SYN flood attack, ICMP flood attack, and HTTP flood attack. The SYN flood attack and HTTP flood attack are directed at three different ports of the central control unit in the dataset, namely ports 80, 443, and 500. The ICMP flood attack was performed three times in a row at different time intervals.

**Fig. 1:**
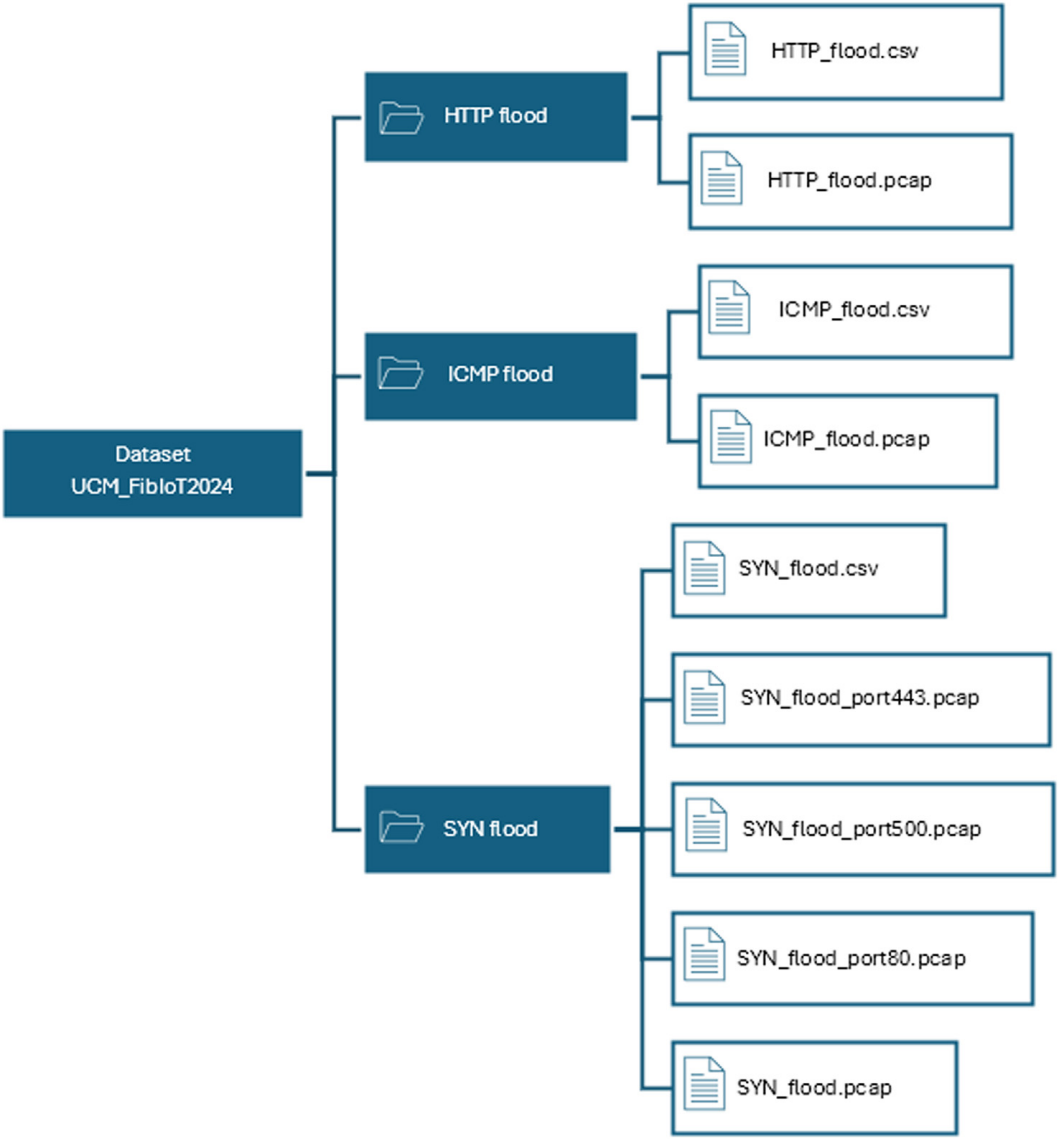
The directory structure of the repository.

The data collected from each attack is organized into .pcap and .csv files, arranged in the folder hierarchy shown in Fig. 1. For ease of handling, the SYN flood attack is further subdivided into smaller files according to the ports on which the attack was executed. Splitting SYN flood PCAP files by port allows for easier and more efficient data processing, as each port represents a different service or application that can be targeted. This allows for faster identification and analysis of specific attack patterns on specific services. To maintain consistency, the dataset also contains the entire SYN flood attack in an undivided format.

The tables in the CSV dataset files have the following columns:

• No. - frame number.
• Time - Date and time of capture (dd.mm.yyyy hh:mm:ss).
• Source - source IP address of the packet.
• Destination - destination IP address of the packet.
• Protocol - protocol type identifying the network protocol used for each packet.
• Length - packet length in bytes.
• Source port - source port of the packet.
• Destination port - the destination port of the packet.

Attacks can be broadly characterized using the following attributes; Table 1, Table 2, Table 3 detail specific attributes for all three types of attacks:

• Start - describes the exact time the attack started (hh:mm:ss.ssss).
• End - describes the exact time of the end of the attack (hh:mm:ss.ssss).
• Start frame - indicates the first packet of the attack.
• End frame - indicates the last packet of the attack.
• Attack Packet Count - indicates the total number of attack packets sent to the device.

**Table 1:** SYN flood attack data in the UCM_FIBIoT2024 dataset (SYN_flood.pcap).

Port	Start Time (hh:mm:ss.ssss)	End Time (hh:mm:ss.ssss)	Start Frame (no.)	End Frame (no.)	Number of attack packets
80	17:36:33.549549	17:40:51.599917	2323	7 442 486	7 033 245
443	17:43:22.156690	17:48:12.993781	7 461 407	15 418 787	7 332 387
500	17:53:28.801293	17:57:13.825988	15 502 247	22 683 151	6 764 677

**Table 2:** ICMP flood attack data in the UCM_FIBIoT2024 dataset (ICMP_flood.pcap).

	Start Time (hh:mm:ss.ssss)	End Time (hh:mm:ss.ssss)	Start Frame (no.)	End Frame (no.)	Number of attack packets
1st attack	18:25:03.731195	18:26:50.412733	4408	4 169 183	4 159 985
2nd attack	18:28:07.882759	18:30:05.568910	4 171 275	8 524 596	4 346 934
3rd attack	18:35:37.262926	18:37:13.803664	8 531 627	11 201 903	2 663 841

**Table 3:** HTTP flood attack data in the UCM_FIBIoT2024 dataset (HTTP_flood.pcap).

Port	Start Time (hh:mm:ss.ssss)	End Time (hh:mm:ss.ssss)	Start Frame (no.)	End Frame (no.)	Number of attack packets
80	18:55:16.422532	18:58:47.677912	1160	2 955 513	149 517
443	19:01:03.396277	19:04:32.442188	2 959 074	9 414 261	721 805
500	19:06:03.272650	19:10:26.912466	9 417 528	10 796 046	172 728

**Fig. 2:**
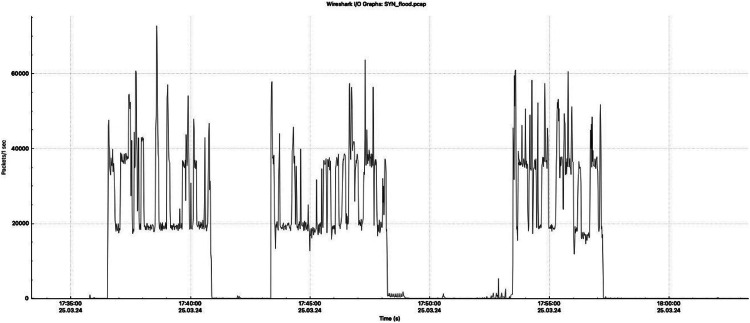
Graph of the number of packets sent per second in SYN flood attacks.

**Fig. 3:**
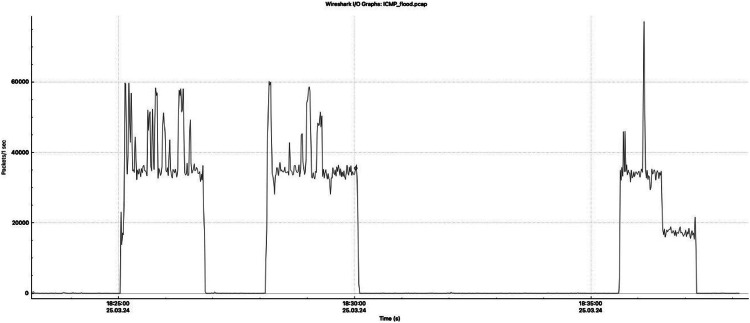
Graph of the number of packets sent per second in ICMP flood attacks.

**Fig. 4:**
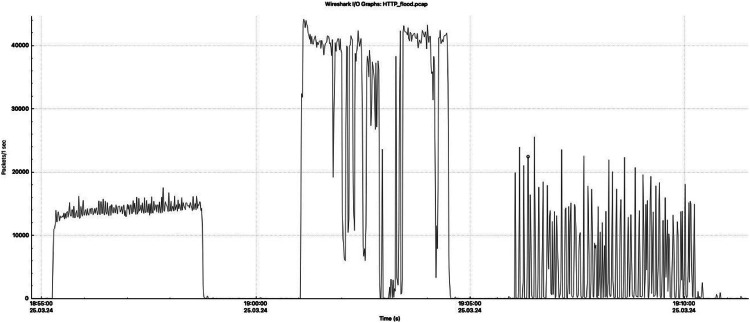
Graph of the number of packets sent per second in HTTP flood attacks.

Fig. 2, Fig. 3, Fig. 4 provide a detailed visualization of the number of packets sent per second during each type of attack recorded in the dataset. These graphs provide a detailed view of the intensity and temporal distribution of SYN flood, ICMP flood, and HTTP flood attacks. These visualizations allow a better understanding of the attack dynamics and identify characteristic patterns of attack packet behavior at different time intervals, which is important for designing effective defense mechanisms. The images were created using the Wireshark tool, which is widely used in network analysis for its ability to monitor and analyze network traffic in detail [9].

The destination address 10.0.1.22 is the address of the Fibaro Home Center 3. Other IP addresses were partially anonymized using generated spoofed public IP addresses.

The dataset also contains normal traffic and is not limited to attack traffic.

The payload has been reduced to 128 bytes due to the possible presence of sensitive information, as there are devices (mobile phone, Fibaro) in the environment that also perform other normal communications.

## 4 Experimental Design, Materials and Methods

A smart home is a system of devices and appliances that can be automated or controlled remotely via a mobile device or other networked device from anywhere with an Internet connection. The Internet connects all the devices in a smart home, allowing the user to control, for example, lighting, temperature, and security systems [10]. A typical home automation system includes a central control unit that integrates and coordinates various home devices such as lighting, electronic doors, fans, and more. The central control unit is connected to the home's local area network via WiFi or Ethernet. It communicates with other IoT devices using low-power protocols such as Zigbee, Z-Wave, or BLE (Bluetooth Low Energy), enabling efficient and automated control of the entire home infrastructure [4].

The smart home, like other digital infrastructures connected to the Internet, is at risk from DDoS attacks. A Distributed Denial of Service (DDoS) attack disrupts network bandwidth by overwhelming all publicly accessible network resources, making them inaccessible. DDoS attacks are more dangerous than traditional DoS attacks because they use multiple attack sources, making them more difficult to detect and mitigate. In a smart home environment, DDoS attacks can be particularly dangerous because devices often use open operating systems that can be easily compromised. In addition, authentication mechanisms in smart homes can pose additional security risks [11].

Three different types of DDoS attacks were implemented in the dataset: SYN flood, ICMP flood, and HTTP flood. Each of these attacks represents a specific method of network intrusion. A SYN flood attack overloads the server with excessive requests to open TCP connections, which leads to exhaustion of its system resources and prevents access by legitimate users. This type of attack attempts to occupy the available connections on a given port, leaving incomplete TCP connections, known as “half-open” connections. The attacker continuously sends new requests until system resources are exhausted, effectively denying connections to legitimate users on the network [12]. An ICMP flood attack is an easy-to-perform attack in which the attacker sends a large number of ICMP echo request packets to overwhelm the target device. This type of DDoS attack causes the target device to become unavailable for legitimate traffic. The attack involves sending massive ICMP (ping) packets that overload the target system, causing it to slow down and become unavailable to legitimate users [13]. An HTTP flood attack is a type of volume attack in which a group of compromised hosts (botnet) send GET or POST requests to flood a Web server. The goal of this attack is to disable the target Web server. In addition to disabling the server, it also causes bandwidth saturation and exhaustion of network device resources. An HTTP flood attack uses legitimate HTTP requests to overwhelm the Web server, causing resource exhaustion and reducing its ability to respond to user requests [14].

The attacks were carried out on the Fibaro Home Center 3 central unit. This device, manufactured by the Polish company Fibaro, which specializes in the production of IoT and home automation devices, represents a universal control system for smart homes. The Fibaro Home Center 3 enables the connection and integration of smart devices with support for wireless communication via various protocols such as Z-Wave, Z-Wave Plus, Zigbee, Wi-Fi, Bluetooth, 433 MHz and 868 MHz. Compared to previous Fibaro models, the Home Center 3 offers higher performance and greater signal range for connected smart devices. The system enables comprehensive home automation and the creation of different scenes. It also offers remote control via a mobile app and integration with Apple Carplay and Android Auto for greater convenience and accessibility [15].

The Fibaro hub used in this study is a central unit for smart home systems that provides various security features comparable to other IoT devices. The Fibaro Home Center 3, like other central units in the IoT ecosystem, provides communication encryption, authentication and device monitoring, which are basic security measures used in smart homes. In addition, as mentioned above, the Fibaro Home Center 3 is compatible with different protocols such as Z-Wave, Zigbee and Wi-Fi, allowing integration with different IoT devices that may have similar or different security features. This compatibility ensures that the principles of protection against cyber-attacks are shared across different platforms and devices within the IoT ecosystem. Based on the above, it can be concluded that the Fibaro Home Center 3 has all the elements that are characteristic of all advanced smart home central units. Another important aspect is that the device is widely distributed in the European region due to the fact that the manufacturer is based in Poland. However, Fibaro products are recognized worldwide and are also used in various regions, including North America and Asia.

**Fig. 5:**
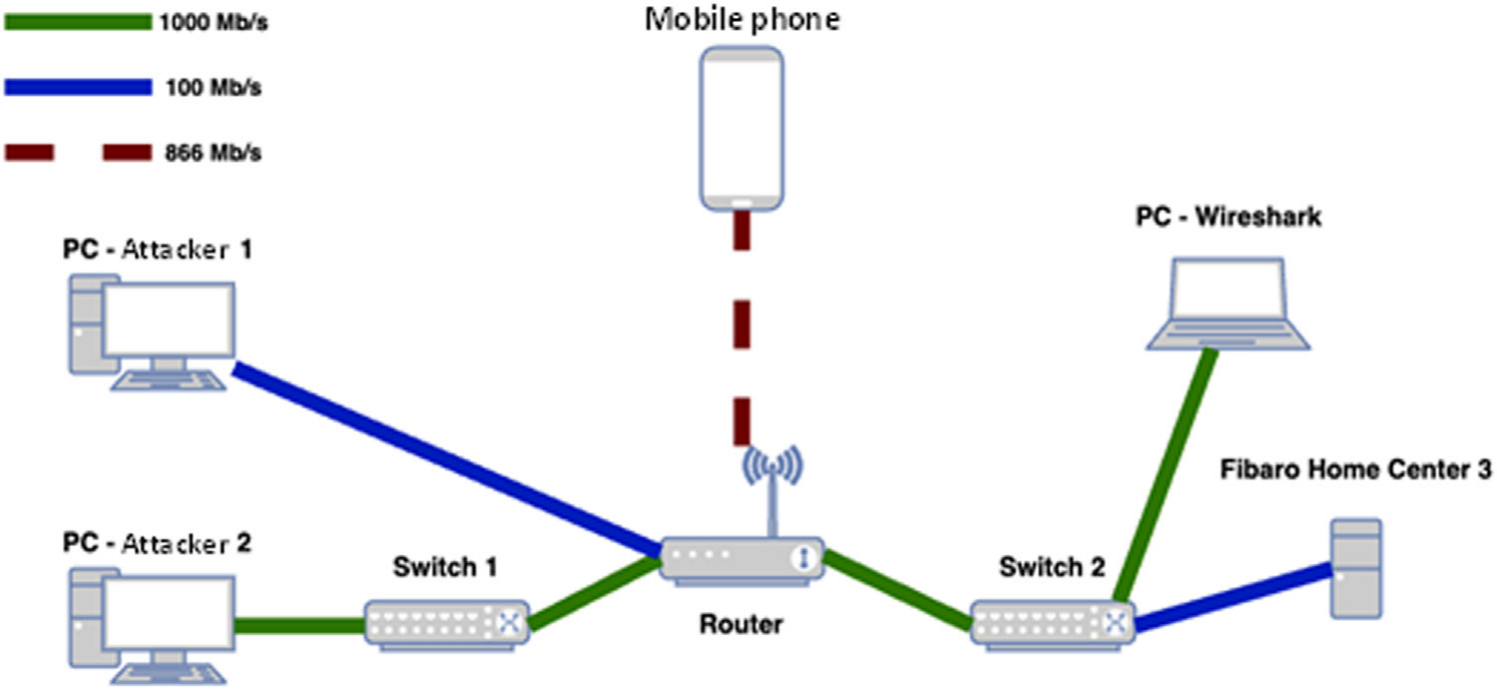
Topology of network connection.

Fig. 5 shows the topology of the devices on the network. Attacker 1 and Attacker 2 are computers running the Kali Linux operating system in VirtualBox. Attacker 1 was connected to the router at 100 Mbps. Attacker 2 was connected through Switch 1 at 1000 Mbps. The Fibaro Home Center 3 unit was connected through Switch 2 at 100 Mbps. A laptop running Wireshark software was also connected through Switch 2 at 1000 Mbps. A mobile phone was connected to the router via Wi-Fi at 866 Mbps. It was used to check the functionality of the mobile application and the alerts from the sensors of the Fibaro Home Center 3 unit during the execution of the attacks.

Flood packets for SYN flood and ICMP flood attacks were generated by two computers using the hping3 tool. The Low Orbit Ion Cannon (LOIC) tool was used to implement the HTTP flood attack.

The hping3 and LOIC tools were used to perform individual attacks; the parameters used to generate the attacks are shown below, with an example of a SYN flood and http flood attack for port 80.

SYN flood: The following command was used to generate SYN flood attacks

*hping3 -S -p 80 –flood –rand-source 192.168.1.22*

ICMP flood: The following command was used to generate ICMP flood attacks

*hping3 −1 –flood –rand-source 192.168.1.22*

HTTP flood: The Low Orbit Ion Cannon (LOIC) tool was used to generate HTTP flood attacks with the following settings

- Target IP address: 192.168.1.22
- Port: 80
- Number of threads: 200
- Time limit: 9001 s

Setting the same parameters for each attack ensures that the data generation can be directly reproduced or that the study can be replicated in different environments.

It should be noted that data recorded on one local network may be consistent with other networks, provided the same network parameters and settings are followed. Network latencies, network topology, and hardware performance are key factors that can affect the results of an attack. Among the most important variables are the type of switches and routers used, and thus the transmission speeds achieved. Especially for flood attacks, transmission speeds are a key parameter. Other important parameters are the security elements of computer networks, such as firewalls and antivirus software, but also the configuration of the network itself and its topology. For a valid comparison, these parameters must be standardized or at least recorded in detail and compared during data analysis. Differences in these parameters can lead to variability in the results, which can affect the interpretation of the data and its applicability to other network environments. Standardization of these conditions is therefore essential to ensure consistency and reliability of results across different experimental settings. Despite the possible variability of the results with different parameters of the test environment, the main benefit of the dataset is the possibility to test developed solutions aimed at improving the security of IoT networks.

## Limitations

One of the main limitations of the dataset is its focus on the Fibaro Home Center 3 device only, which may affect the ability of the results to be generalized to smart home central control devices. Another limitation is that the tests were performed on a limited number of devices and with specific hping3 and LOIC tools.

The simulated attacks focused only on three types of DDoS attacks (SYN flood, ICMP flood, and HTTP flood) and do not represent the full range of possible DDoS attacks on IoT devices.

## Ethics Statement

This work does not involve human subjects, animal experiments, or any data collected from social media platforms. In addition, the data is anonymized, and the payload of the packets is removed in order to prevent the user confidentiality.

## Credit Author Statement

**Ladislav Huraj:** Methodology, Software, Supervision, Validation, Resources, Data curation, Writing – original draft. **Marek Šimon:** Conceptualization, Methodology, Validation, Investigation, Project administration, Writing – review & editing. **Jakub Lietava:** Conceptualization, Methodology, Validation, Investigation, Writing – original draft.

## Data Availability

Mendeley DataUCM_FibIoT2024 (Original data).
